# Zika virus and brain cancer: Can Zika be an effective treatment for brain cancer? A systematic review

**DOI:** 10.18632/oncotarget.28647

**Published:** 2024-09-30

**Authors:** Mateus Gonçalves de Sena Barbosa, Beatriz Rodrigues Messias, Rafael Trindade Tatit, Maycon Cristian Gomes de Paula, Valdecir Boeno Spenazato Júnior, Maria Gabriella Borges Braga, Caio Vinícius Marcolino Santos, Luiza D'Ottaviano Cobos, Vinícius Otávio da Silva, Eberval Gadelha Figueiredo, Nicollas Nunes Rabelo, Bipin Chaurasia

**Affiliations:** ^1^Department of Neurosurgery, Atenas University Center, Passos, Minas Gerais, Brazil; ^2^Hospital Israelita Albert Einstein, University of Israelita de Ciências da Saúde Albert Einstein, São Paulo, Brazil; ^3^Department of Neurosurgery, University of Sapucaí Valley, Pouso Alegre, Minas Gerais, Brazil; ^4^Department of Neurosurgery, Atenas University Center, Sete Lagoas, Minas Gerais, Brazil; ^5^Department of Neurosurgery, Nove de Julho University, Campus Vergueiro, São Paulo, Brazil; ^6^Department of Neurosurgery, José do Rosário Vellano University, Alfenas, Minas Gerais, Brazil; ^7^Division of Neurosurgery, School of Medicine-University of São Paulo (FMUSP), Hospital das Clínicas/FMUSP, São Paulo, Brazil; ^8^Department of Neurosurgery, Neurosurgery Clinic, Birgunj, Nepal

**Keywords:** Zika, neurotropism, glioblastoma, glioma, brain tumor

## Abstract

Introduction: Many studies have highlighted the use of oncolytic viruses as a new class of therapeutic agents for central nervous system (CNS) tumors, especially glioblastomas (GMB). Zika Virus (ZIKV) proteins targeted to specific stem cells have been studied *in vitro* and animal models with promising results.

Materials and Methods: A systematic review was evaluated the efficacy and safety of the ZIKV use for CNS tumors treatment. Data were extracted and the *in vivo* studies were evaluated using the Robins-I tool. We assessed bias in each study using criteria such as selection bias, performance bias, detection bias, attrition bias, reporting bias, and others. According to Cochrane guidelines, bias was classified as high, low, or uncertain. High bias occurred when studies did not meet the criteria. Low bias was assigned when criteria were clearly met. Uncertain bias reflected insufficient information for a clear classification.

Results: The 14 included studies shown that ZIKV reduced cell viability or inhibited the growth, proliferation of glioma stem cells (GSCs), and Bcl2 expression - which could potentially enhance the effect of chemotherapy/radiotherapy; caused cytopathic effects, induced tumor cell damage, manifested oncolytic properties, and even selectively safely killed GSCs; ultimately, it led to significant tumor remission and enhanced long-term survival through enhanced T-cell response.

Conclusions: Although current evidence suggests ZIKV as a promising treatment for CNS tumors and may improve survival when combined with surgery and radiotherapy. Despite limited human evidence, it shows potential benefits. Further research is needed to confirm safety, efficacy, and optimize treatment in humans.

## INTRODUCTION

Central nervous system (CNS) tumors result from irregular cellular growth in the brain and spinal cord, accompanied by neurological symptoms. According to “Global Cancer Statistics 2020,” CNS tumors significantly impact global cancer burden, with over 300,000 new cases and a mortality rate of 2.5%. Incidence and mortality vary geographically, highlighting the need for localized public health strategies. Projections indicate an increase in CNS tumors by 2040, stressing the need for improved prevention and treatment research. The prognosis of brain tumors depends on the type of tumor and possible treatments involve surgery, associated with radio and/or chemotherapy [1–5].

Glioblastoma multiforme (GBM) is the most aggressive and common primary brain tumor in adults, originating from astrocytes. It grows rapidly, is highly invasive, and often recurs post-treatment. Symptoms vary based on tumor location and include headaches, seizures, and cognitive deficits. Despite treatments like surgery, radiotherapy, and chemotherapy, GBM has a poor prognosis, with median survival of 12 to 15 months. The tumor’s resistance to conventional therapies is due to its heterogeneity and adaptability. Innovative approaches, such as using the Zika virus (ZIKV) protein NS5, are being explored to improve outcomes for GBM patients [6–10].

ZIKV, an arbovirus from the Flaviviridae family, gained attention during the 2015 epidemic due to its neurological impact on fetuses, causing microcephaly and other anomalies. Its neurotropism makes it a candidate for treating CNS tumors [5, 6]. Studies suggest ZIKV can reduce tumor cell proliferation, induce apoptosis, and enhance immune responses against CNS tumors. Specific ZIKV viral proteins, particularly those with tropism for GBM stem cells, have shown promise in treatment [11–14].

ZIKV’s oncolytic properties against GBM involve multiple mechanisms: strong tropism for neural progenitor cells, induction of apoptosis via Caspase-3 activation [15], inhibition of glioma stem cell tumorigenicity by NS5 [4], and modulation of cellular signaling proteins such as NOTCH and NUMB [7]. ZIKV also downregulates Bcl-2, promoting further cell death in glioblastoma cells, highlighting its potential as an effective oncolytic therapy [16].

The objective of this systematic review is to elucidate the potential use of Zika virus (ZIKV) and its fragments in the treatment of CNS tumors, particularly GBM. The review synthesizes existing literature to evaluate ZIKV’s effectiveness in reducing tumor cell proliferation, inducing apoptosis, and augmenting immune responses against CNS tumors.

## RESULTS

### Search strategy results

**Figure 1:**
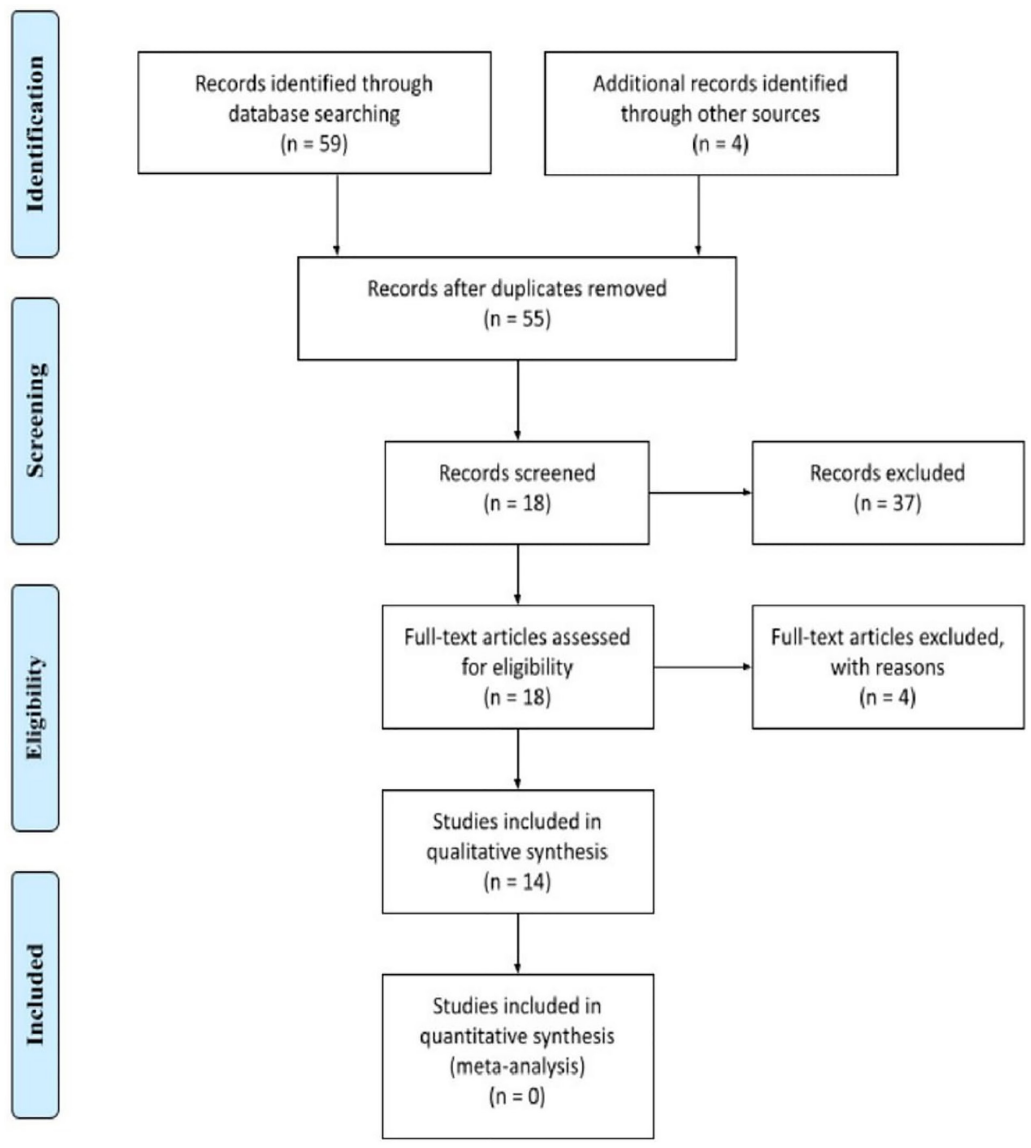
Flowchart: Represent identification, screening, eligibility and Inclusion and exclusion criteria of this systematic review.

Applying the search strategy previously described, 63 records were identified, from the following sources: PubMed, Embase and Scopus. After the exclusion of duplicates, 55 articles were screened. Thirty-seven studies do not meet the inclusion criteria, hence being excluded after initial evaluation of title and abstract. The 18 remaining papers received an assessment through full-text reviewing, giving the potential eligibility for this systematic review. Of these, 4 were excluded for the following reasons: review article [1], focus on replicative mechanisms of ZIKV [1], parallel between ZIKV infection and sandfly fever Turkey virus and assessment of photobiomodulation as a therapy against ZIKV infection [1]. Following this process, 14 studies were included into this systematic review, all in English. The search strategy is summarized in Figure 1.

### Included studies

The main features of included papers are summarized in Table 1. Out of the 14 experimental studies assessed, 5 were *in vitro* and 9, *in vivo*/*in vitro*. Upon assessment of the articles, significant heterogeneity was detected in the investigated outcomes, highlighting different biomarkers and metabolic/signaling pathways. Applying the type of conducted study as a criterion, for didactic purposes, the articles included in this review can be divided into three subgroups: *in vitro* [1, 5], *in vivo* e *in vitro*/*in vivo* [2–4].

**Table 1:** Summary of the main *in vitro*/*in vivo* studies (2017–2021) investigating the oncolytic effects of Zika virus (ZIKV) in CNS tumors

Study	References	Title of the study	ZIKV strain	Cell lineage	Biomarker	Outcomes
1.	Chen Qi, Wu Jin, Ye Qing, et al. 2018 [9]	Treatment of Human Glioblastoma with a Live Attenuated Zika Virus Vaccine Candidate	FSS 13025/GZ01	GSCs specimens 387 and 4121	–	ZIKV-LAV was shown to be safe and significantly intracerebral tumor growth and reduced animal survival by selectively killing GSCs within the tumor
2.	Crane AT, Chrostek MR, Krishna VD, et al. 2020 [10]	Zika virus-based immunotherapy enhances long-term survival of rodents with brain tumors through upregulation of memory T-cells	ZIKV H/PF/2013	GL261 GBM cells; GS-9L glioma cell line	–	ZIKV immunotherapy could be an adjuvant to tumor vaccines to intensify long-term survival, through enhanced T-cell response
3.	Dabaja MZ, Lima EO, Oliveira DN, et al. 2018 [6]	Metabolic alterations induced by attenuated Zika virus in glioblastoma cells	ZIKV^BR^	U-251 GBM cells	Phospholipids¹, chlorinated metabolite², phosphatidylinositol-3-phosphate	ZVp might be an alternative treatment for GBM, given the cytopathic effects and cell damage induced on neural tumor cells
4.	Iannolo G, Sciuto MR, Cuscino N, et al. 2019 [11]	Zika virus infection induces MiR34c expression in glioblastoma stem cells: new perspectives for brain tumor treatments	ZIKV H/PF/2013	GSCs U87MG and T98G	CD133, SOX-2, Musashi-1, and nestin	ZIKV infection induced miR34c expression and its overexpression reproduced an effect equivalent to that of infection. Mir34c can inhibit GSCs and reduce Bcl2, which could potentially enhance the effect of chemotherapy/radiotherapy.
5.	Kaid C, Goulart E, Caires-Júnior LC, et al. 2018 [7]	Zika Virus Selectively Kills Aggressive Human Embryonal CNS Tumor Cells *In Vitro* and *In Vivo*	ZIKV^BR^	Embryonal CNS tumor cell lines: DAOY, USP13-MED, USP7-ATRT	Wnt/β-catenin pathway	ZIKV has oncolytic properties and specifically targeted stem-like cancer cells from embryonal CNS tumors
6.	Kaid C, Madi R, Astray R, et al. 2020 [2]	Safety, Tumor Reduction, and Clinical Impact of Zika Virus Injection in Dogs with Advanced-Stage Brain Tumors	ZIKV^BR^	CNS primary tumor with neural origin, excluding meningioma and other non-neural tumors	anti-ZIKV NS2B antibody	Shown for the first time significant CNS tumor remission following ZIKVBR intrathecal injections in two dogs bearing spontaneous intracranial tumors with an absence of clinical side effects associated with ZIKV infection.
7.	Li H, Hu Y, Huang J, et al. 2019 [4]	Zika virus NS5 protein inhibits cell growth and invasion of glioma	PRVABC59	HEK293T, U87 and GL261 glioma cell lines	NS5	NS5 viral protein inhibited cell growth and proliferation and tumorsphere formation
8.	Li M, Zhang D, Li C, et al. 2020 [12]	Characterization of Zika Virus Endocytic Pathways in Human Glioblastoma Cells	kv963796	Glioblastoma T98G cells	clathrin heavy chain	Viruses penetrate cells by various mechanisms, including fusion with the cell membrane or entering by receptor-mediated endocytosis.Clathrin-mediated endocytosis is the most frequently used pathway. ZIKV can enter T98G cells through not only clathrin-dependent but also clathrin-independent pathways
9.	Lima E, Guerreiro T, Melo C, et al. 2017 [13]	MALDI-Imaging detects endogenous Digoxin in glioblastoma cells infected by Zika virus – would it be the oncolytic key?	ZIKV^BR^	M059J GBM cells	Digoxin	ZIKV induced cytopathic effects, associated with endogenous digoxin synthesis, at GBM cells
10.	Luplertlop N, Suwanmanee S, Muangkaew W, et al. 2017 [14]	The impact of Zika virus infection on human neuroblastoma (SH-SY5Y) cell line	SV0127/14 and SV0010/15	human neuroblastoma cell line (SH-SY5Y)	–	–
11.	Mazar J, Li Y, Rosado A, et al. 2018 [15]	Zika virus as an oncolytic treatment of human neuroblastomacells requires CD24	PRVABC59	Neuroblastoma MYCN and non-MYCN	NS1	ZIKV infection reduces cell viability. However, the permiveness to zika virus depends on CD24 expression. It occours mainly on high metabolic activity progenitors, not having this effect on differentiated cells
12.	Nair S, Mazzoccoli L, Jash A, et al. 2021 [3]	Zika virus oncolytic activity requires CD8+ T cells and is boosted by immune checkpoint blockade	ZIKV-Dakar	GL261 and CT2A GBM cells	CD8-depleting antibodies, isotype control IgG2b, antibodies against PD-1, IgG2a control	Histological analysis revealed comparable tumor sizes between the ZIKV and PBS groups at day 14 after tumor implantation (7 days after ZIKV treatment) but a decrease in tumor size 1 week later at day 21 after tumor implantation (14 days after ZIKV treatment) in response to ZIKV treatment. It was observed infiltration of immune cells in the tumor microenvironment at days 14 and 21 after tumor implantation in animals treated with ZIKV. ZIKV treatment also increased the tumor-associated myeloid cell response in the tumor bed, particularly the monocyte and microglia populations.
13.	Trus I, Berube N, Jiang P, et al. 2020 [16]	Zika Virus with Increased CpG Dinucleotide Frequencies Shows Oncolytic Activity in Glioblastoma Stem Cells	PRVABC59	C6/36 cells	–	*In vitro*: reduced infection kinetics in nonmalignant brain cells but high infectivity and oncolytic activity in GSCs. *In vivo*: efficiently replicated with a significant reduction of tumor growth
14.	Zhu Z, Mesci P, Bernatchez J, et al. 2020 [5]	Zika Virus Targets Glioblastoma Stem Cells through a SOX2-Integrin avb5 Axis	H/PAN/ 2016/BEI-259634 and PRVABC59	293FT Cell Line, ENSA (ENS-tem-A), NSC11, NM53, NM55, NM177, NPC C4-7, hNP1 (STEMEZ hNP1) and H1 ESC	–	avb5 integrin was shown to be a functional marker of cancer stem cells essential for maintenance of GBM and ZIKV infection

Abbreviations: CNS: central nervous system; GBM: glioblastoma; MALDI: matrix laser desorption/ionization mass spectrometry imaging; ZVp: attenuated ZIKV prototype. Lysophosphatidic acid, oxidized phosphatidylserine and simple phosphatidylserine; 5-methyltetrahydropteroyltri-L-glutamate.

### Outcomes

#### *In vitro* studies

Aiming to answer the question “what would be the effects of ZIKV infection on neural tumor cells?”, Lima et al. [13] conducted an experimental study, in which M059J GBM cells were divided into ZIKV group, submitted to viral inoculation, and control group. Microscopic examination was performed 24- and 48-hours post-infection, which showed mild cytopathic effects induced by ZIKV at GBM cells at the first time point of analysis, but evidence of pronounced cell death because of ZIKV infection, when compared with CT-group, was found at 48-hours post-infection. For the evaluation of metabolomic changes associated with ZIKV in GBM cells, both cell cultures were assessed by MALDI-MSI, evidencing a difference in metabolite compositions between infected and non-infected GBM cells. The statistical analysis provided evidence that digoxin, a cardiac glycoside, was significantly more expressed in ZIKV group. Therefore, this study suggests that genetically modified ZIKV might be an alternative for GBM management, through the synthesis of Digoxin, associated with cytopathic effects.

Aiming to investigate the real impact of ZIKV on human adult neuronal cells, Luplertlop et al. [14] shown the presence of ZIKV particle inside the nucleus of infected SH-SY5Y neuroblastoma cells, and the loss of nucleus membrane indicates that they might cross this membrane for multiplication and destroy it [14].

In 2018, Dabaja et al. [6] carried out the evaluation of metabolic alterations induced by ZIKV in GBM cells, developing a attenuated ZIKV prototype (ZVp) with viral fragments encapsulated into bacterial outer membrane vesicles (OMV). U-251 GBM cells were divided into four subgroups: CT-group, empty OMV, ZIKV and ZVp. Similar to the findings of Lima et al. [13], microscopic analysis showed mild cytopathic effects induced by ZVp 24 hours after the infection. Alterations were even slighter in OMV and ZIKV groups and no changes were observed in CT group. At the second timepoint (48 hours), the mild effects turned into substantial difference in cell count, with ZVp group presenting fewer neural tumor cells when compared to other groups. This study also highlights increased cell damage and TNF-alpha expression in the ZVp group, which indicates GBM cell death. In addition, a metabolomics comparison between ZVp and CT groups was performed. Different biomarkers were elected, including three phospholipids, a chlorinated metabolite and phosphatidylinositol-3-phosphate, which may be produced in the oxidative environment induced by ZVp and hence reflect cell death. All these findings bring light to ZVp as a feasible alternative for GBM management, encouraging further *in vivo* studies.

In a similar way, Mazar et al. [15] aimed to study metabolic issues on the ZIKV action on Neuroblastoma cells. They demonstrated that the cell viability decrease due to ZIKV infection occurs mainly on high metabolic activity progenitors, not having this effect on differentiated cells. However, the vulnerability to zika virus depends on CD24 expression. Therefore, they proposed that therapeutic ZIKV infection of individuals with CD24-positive tumors have a better prognosis, been a good prognostic marker in this treatment [15].

It is important to understand the endocytic pathways of the ZIKV, using that for further therapeutic indications and development of new treatment strategies. Li M et al. [12] search this pathway on GBM T98G cells. They found that Clathrin-mediated endocytosis is the most frequently used pathway. ZIKV can enter T98G cells through not only clathrin-dependent but also clathrin-independent pathways. Caveola-mediated pathway have an important role in the entry of ZIKV into T98G cells. Depletion or sequestration of cholesterol from the membrane by MβCD or filipin inhibited the ZIKV entry into T98G0 cells [12].

#### *In vivo/in vitro* studies

To assess which ZIKV non-structural protein (NS1, NS3, NS4B, NS5) is responsible for inhibit tumor cell growth, Li et al. [4] conducted an *in vivo/in vitro* experiment in 2019. Four genes related to the previously quoted proteins were inserted in U87 glioma cells and the efficiency of transfection was confirmed by immunofluorescence. Next, the investigators found that NS5 significantly inhibited neural tumor cells proliferation when compared to other viral proteins, as demonstrated by lower expression of Ki-67. This effect was also shown in tumorsphere decrease, both in size and in number. NS5, besides reducing cell proliferation, also suppressed migration and invasion of U87 lineage. *In vivo,* outcomes in mouse GL261 glioma cells were similar, with significantly lesser formation of tumorpheres and higher survival time when compared to CT group (*P* < 0.05). Thus, Li et al. [4] successfully showed that expression of NS5 ZIKV protein inhibits tumorigenicity *in vitro* and *in vivo*.

More recently, Crane et al. [10] investigated ZIKV as a therapeutical option for GBM, developing an *in vivo* experiment. First, the authors proved, as suggested by previously studies, that GL261 GBM cells are prone to ZIKV infection *in vitro*, given that an increase in virus particles was observed among infected cells. To assess if ZIKV infection could improve overall survival (OS) *in vivo*, mice with GL261 tumor lineage received intracranial (i.c.) injection of ZIKV. There were no significant differences in OS when compared to non-treated mice. The same outcomes were observed in rats with 9L glioma cell line, suggesting that i.c. infection with ZIKV, alone, is not suitable for glioma/GBM management, different to other authors findings. Next, the investigators focused on ZIKV infection as a co-therapy, along with a vaccine-based treatment. Therefore, mice with GL261 tumor lineage were subcutaneously vaccinated with irradiated GL261 cells infected with ZIKV. The study also proceeded with i.c. injection ZIKV. OS in the group comprised of mice treated with i.c. ZIKV plus vaccine was not significantly higher than the CT-group or vaccine-group alone. Although no difference in the OS was noted, long-term survivors received another i.c. tumor injection or i.c. saline to assess the immune response. Mice in the tumor rechallenge group presented an increase of total T-cells and CD4+ T cells, which might suggest that treatment with i.c. ZIKV plus vaccine strengthens immune response.

Crane et al. [10] also investigated if i.c. injection of ZIKV 14 days after vaccine therapy could improve OS, given that it is expected to have a peak of T-cells 10 days following vaccination. After vaccination, the GL261-mice were then divided into three subgroups: i.c injection of attenuated ZIKV on day 0, i.c injection of aZIKV on day 14 and i.c. injection of vaccine on day 14. The second group showed an improvement of OS, relative to untreated mice (*P* < 0.001). In addition, the group of mice treated with subcutaneous vaccination plus i.c vaccine presented the highest number of long-term survivors (*P* < 0.001). Hence, this study suggests that ZIKV can be used as an adjuvant therapy along with vaccination to improve long-term survival of mice with GBM/glioma, as a consequence of CD4+ T-cell response and production of memory T-cells capable of respond selectively to tumor cells.

Aiming to show that ZIKV reshapes the immune response, Nair et al. [3] implanted glioma cells in the right hemisphere of mice. After its growth, they infected with ZIKV increasing median survival, and the long-term survival rates from approximately 10% to 63%. Further, they engineered a ZIKV Dakar clone (Δ10 3′-UTR ZIKV) that produced short subgenomic flaviviral RNAt RNA species that antagonizes cell-intrinsic innate immune responses. This, associated to anti–PD-1 immunotherapy prolonged median survival to 33.5 days after tumor implantation, and the survivor rate increased from 0% to approximately 40% in the combination treatment group, suggesting that this response was driven by CD8 T cells [3].

Kaid et al. [7], in turn, aimed to understand how stem-like cancer cells from human embryonal CNS tumor behave in face of ZIKV infection. For this, the study focused on assess three embryonal CNS tumor lineages (DAOY, USP13-MED and USP7-ATRT), as well as three non-CNS tumor cell lines from breast, colorectal and prostate cancer. All the six cell lines were infected with ZIKV, in order to evaluate *in vitro* oncolytic effects of ZIKV infection. 72-hours after the infection, the investigators observed cell death and/or growth reduction in all the CNS tumor lineages, although infection of DAOY was less pronounced when compared to USP13-MED and USP7-ATRT. Flow cytometry analysis was performed and showed an increase in the population of PI-positive CNS tumor cell lines as a consequence of ZIKV infection, suggesting cell death through rupture of plasma membrane. It was also stated that ZIKV infection interfered with CNS tumorspheres, mainly CNS embryonal tumorspheres. However, mild to no effect in oncolytic properties and tumorsphere disruption was seen on non-CNS tumor cell lines. Based on these findings, the authors proposed a selective ZIKV-infection and cell death of CNS tumor cells, when compared to normal CNS stem cells and other tumor cell lines (prostate, breast, colorectal).Two years later, the same author shown for the first time significant CNS tumor remission following ZIKV^BR^ intrathecal injections in two dogs bearing spontaneous intracranial tumors with an absence of clinical side effects associated with ZIKV infection [2].

Next, an *in vivo* study was conducted with a intracerebroventricular injection of ZIKV in BALB/c nude mice after period of CNS tumor establishment (1 to 2 weeks for DAOY, USP13-MED and USP7-ATRT cell lines). In this study, ZIKV was shown to induce remission in 20 of 29 animals within the experimental group, with complete remission in 7 mice. When compared to sham group, OS of USP7-ATRT tumor-bearing mice treated with ZIKV infection was statistically increased (*P* = 0.0046) and 60% of the group had complete metastatic remission (*n* = 3) [9]. Reduction of tumor growth ratio in USP7-ATRT and USP13-MED was also observed, even though DAOY cell line had a poor response to ZIKV infection, which fits *in vitro* findings. In addition, the study suggests that Wnt/β-catenin pathway may be involved in cell death associated with ZIKV infection, given that USP7-ATRT, cell line with best outcomes, had shown hyperactivity of this specific pathway [9].

### Risk of bias assessment

**Figure 2:**
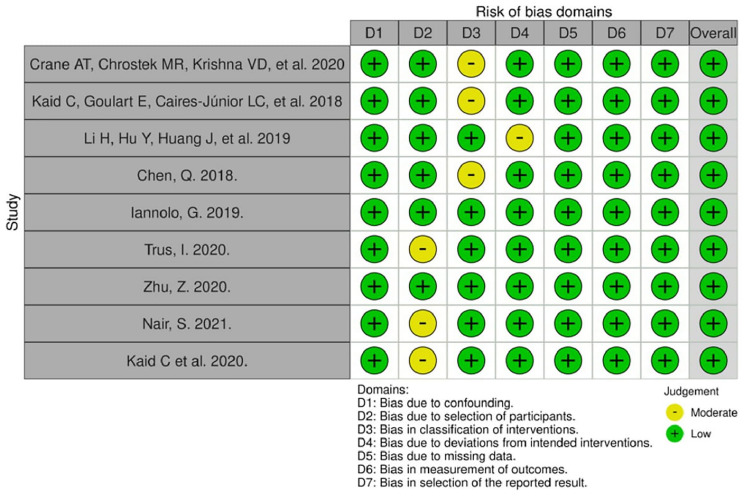
Risk of bias for *in vivo* studies (Robins-I tool).

The quality assessment results are presented in Figure 2. Only *in vivo* studies were subjected to risk of bias analysis, due to lack of reliable and universally accepted assessment tools to investigate *in vitro* studies. Overall, the risk of bias was judged to be low. A moderate risk was more prevalent in selection of participants and classification of interventions, given that all of the *in vivo* studies were conducted on animals, hence the concepts of selection and blinding are controversial. The majority of bias domains found in this assessment can be considered inherent to the study design.

## DISCUSSION

Treatments for brain cancer using pathogens, an oncolytic viral therapy, have been widely researched [4]. There is a growing focus on the use of ZIKV as a potential pathogen for this type of intervention. This article sought to evaluate the behavior of a tumor, GBM, in the presence of this virus [13].

The detailed mechanisms of ZIKV’s pathophysiology are the focus of studies that have already identified the virus’s tropism for neuronal progenitor cells. Therefore, cell apoptosis is observed through Caspase-3 apoptosis, a change in the cell cycle, which explains the alteration found in most newborns during gestation. In adults whose nervous systems are fully developed, studies suggest that ZIKV does not exhibit the same neurotropic effects observed in developing nervous systems [13]. Most patients infected with ZIKV are asymptomatic; those who do exhibit symptoms typically experience conjunctivitis, fever, and rash, with a self-limited infection where a small portion develops paralysis and neuropathy [12, 14].

Therefore, GBM, a common cancer with a grim prognosis and low survival rates, has been discussed with special emphasis. It is characterized by its origin in neuronal stem cells, which are precisely the target of the virus, i.e., affecting the same type of cell [3]. Consequently, it is observed that there will be interference in the neuronal ability to generate action potentials, especially for inactive cells, causing tumor reduction.

ZIKV presents a non-structural protein (NS5), which significantly inhibits the tumorigenicity of glioma stem cells, reducing their proliferation [4]. NS5 demonstrated inhibition of tumor growth in GL261 and U87 (glioblastoma cell lines) in animal samples, through the mediation of apoptosis pathways and inhibition of cell migration and invasion, as these cells were found to be permissive to ZIKV infection [4, 10]. Additionally, cardiac glycoside molecules, such as digoxin, which were identified early in ZIKV infection, showed good results in patients with breast cancer, neuroblastoma, and melanoma [13], and also increased p53 activity, leading to DNA damage [6]. However, the complete mechanism is still not fully elucidated [4].

In a study conducted by Kaid et al. 2018 [7], it was seen that the virus could act positively in other types of cancer, such as breast, prostate, medulloblastoma, and rhabdoid teratoid tumor, however they obtained specific good results for the central nervous system, especially the rhabdoid teratoid tumor, as it originates from stem and neuroprogenitor cells [7]. Another research revealed the significant efficacy of neuroblastoma treatment, where the virus eliminated most tumor cells in a few days [14].

Furthermore, GBM remission was observed in mice that survived vaccination with cells previously infected with ZIKV and through intracranial injections of live attenuated virus or cells that were previously infected. In this group, immunization from the generation of memory T cells, with significant survival, was achieved. Thus, ZIKV can contribute to the development of vaccines [10]. Another study shows that, with just one intracerebroventricular injection in mice, there was a reduction in viral load, increased survival, and lower incidence of remission and metastasis [7].

In a study that observed high cost-effectiveness when applying the Brazilian attenuated ZIKV prototype with encapsulated fragments of *Neisseria meningitidis* (ZVp), which would mainly act on GBMs but also influence other tumors [6]. Chen et al. 2018, through the analysis of the safety and efficacy of a live attenuated Zika virus vaccine (ZIKV-LAV) for the treatment of human GBM in an orthotopic model, found that ZIKV-LAV impairs GBM formation, prolongs animal survival, has tumoricidal activity in GBM cells, and preferentially infects and kills glioma stem cells, thus presenting an excellent safety profile necessary for brain virotherapy and with potent oncolytic efficacy [9].

The human neuroblastoma cell line (SH-SY5Y) was found to be permissive to ZIKV infection and replication and can be used as an *in vitro* model of adult human neuronal cells to further elucidate ZIKV biology and neuropathology [14]. Another experimental study (dogs) demonstrated the absence of negative side effects after Brazilian ZIKV injections, but also tumor reduction in immunocompetent dogs with spontaneous intracranial tumors, improvement of neurological symptoms, and prolonged survival [2].

According to a study conducted by Zhu et al. 2020 [5], when evaluating GSCs, oncolytic activity was identified in cells infected by ZIKV, with reduced tumor growth. Furthermore, the SOX2 gene, a transcription factor responsible for inducing pluripotency in neural tissue and glioblastoma stem cells, was highlighted. Their analysis concluded that the SOX2 gene is also associated with immune response suppression, resulting in higher infection of GSCs by ZIKV [5].

Zhu et al. 2020 [5], also clarified that ZIKV infectivity in GBM tumor cells depends on the expression of alpha-v-beta-5 integrins in the neoplastic tissue of GSCs. These integrins are important receptors for ZIKV infection, and when inhibited, infection rates decrease. Additionally, alpha-v-beta-5 integrins regulate the cytotoxicity of tumor cells. Their inhibition reduces GSC viability and interferes with the survival of these cells [5].

ZIKV infection also induced inhibition of the development of genes responsible for tumor cell maintenance and proliferation, such as NOTCH (a signaling protein that regulates cellular development and maintenance of stem cells) and NUMB (a negative regulator protein of the NOTCH pathway). Negative modulation of NUMB induces proteasome-dependent degradation of p73. It was found that p73 confers an invasive phenotype to glioblastoma cells, and its deletion impairs invasion and chemoresistance in animal models and glioblastoma patients, prolonging survival. Additionally, ZIKV reduces the expression of Bcl2, a protein that inhibits apoptosis and regulates cell death mechanisms. Thus, the reduction of Bcl2 and NUMB decreases AKT phosphorylation and increases the apoptotic response in glioblastoma cell lines, highlighting the importance of ZIKV as a potential oncolytic therapy for glioblastoma treatment [11].

An analysis conducted on glioblastoma stem cells (GSCs) showed that the induction of miR34c production, an important microRNA in regulating cellular functions, apoptosis, and differentiation, reduced the growth of these cells. This induction also regulated the expression of Bcl2 and NUMB, mimicking the effect observed in ZIKV infection. The answer obtained was a reduction in tumor growth, promoting oncolytic activity in GBM treatment [11].

Nevertheless, the ability of GBM to resist ZIKV activity *in vivo* still needs to be studied [16]. It was observed that some GSC lines *in vitro*, derived from CpG recoding in the ZIKV viral genome and the use of CpG dinucleotide implementation technology for the development of oncolytic candidates, may have different results in oncolytic response. Despite this, these lines showed a considerable reduction in infectious titers and the number of cells infected by ZIKV. This dissonance between different CpG-recoded variants demonstrates that the oncolytic activity of a virus can be adjusted according to the number of newly introduced CpG dinucleotides in a viral genome. Therefore, oncolytic therapy still needs to better understand the behavior among CpG-recoded viruses, the tumor, the tumor environment, and host responses to become more effective [11, 16].

Although the use of the Zika virus (ZIKV) as a therapy for glioblastoma multiforme (GBM) shows potential, it presents several significant disadvantages and risks. There are concerns about unknown side effects in humans, including possible complications in people with compromised immune systems, as well as the risk of uncontrolled infection and viral reactivation. Adverse immune responses are another concern, potentially causing inflammation or other harmful reactions. The efficacy of the treatment may also vary among patients due to the dependence on the expression of specific receptors, such as integrins alpha-v-beta-5, on tumor cells [5].

Moreover, there is the risk of developing viral resistance, which could reduce the treatment’s effectiveness over time. There are uncertainties about the potential long-term neurotoxic effects of ZIKV on the central nervous system of adults. Ethical and regulatory challenges are also significant, as the use of a pathogen as an oncological treatment raises ethical questions and may face regulatory barriers before being approved for clinical use. Therefore, while ZIKV presents a promising path, it is crucial to address these disadvantages and risks with ongoing research and a cautious approach.

## MATERIALS AND METHODS

### Study design and identification

This is a systematic review, based on the guidelines of preferred reporting items for systematic reviews and meta-analyses (PRISMA) [17–19]. A systematic and comprehensive literature review was performed from MEDLINE, EMBASE, Cochrane Central Register of Controlled Trials, Web of Science and SciELO. The search was performed with terms associated to: “brain tumor”, “brain cancer”, “brain neoplasm”, “glioma”, “glioblastoma”, “neuroblastoma”, “stem cells”, “oncology”, “zika virus”, “oncolytic”, “oncolysis”, “treatment”, “therapy”, “immunotherapy”, “immunology”, “approach”, “outcome”, “outcome”, “vaccine”, “anticancer”, “digoxin” and “follow-up”. Each article and its respective references were obtained in full and carefully analyzed. Protocol and registration code: PROSPERO 2022 CRD42022338809.

### Eligibility criteria

Articles that presented scientific evidence on the presence or absence of the oncolytic capacity of the Zika virus (ZIKV) against brain tumors, and/or the effectiveness or inefficacy of this virus in combating brain tumors, were included. This encompassed studies that provided clear data on the impact of ZIKV on brain tumor cells, whether through *in vitro* experiments, *in vivo* studies, or clinical trials. Both qualitative and quantitative primary research (including primary studies) and secondary research were included if they were available online in full-text format in English, Spanish, or Portuguese. Studies were selected based on their relevance and contribution to understanding the use of ZIKV in the treatment of CNS tumors.

To ensure a comprehensive review, additional relevant studies were identified in the references section of the included articles. A manual search using the “snowball” method was also conducted to find and include relevant and reliable gray literature. This gray literature was subjected to the same selection criteria to ensure consistency and reliability in the review process. By applying these criteria, the review aimed to compile a robust and thorough body of evidence regarding the oncolytic potential of ZIKV in the treatment of brain tumors.

### Exclusion criteria

Articles were excluded if they were narrative or integrative reviews, monographs, or letters to the editor due to their lack of original empirical data and limited contribution to the evidence base. Studies were also excluded if they had methodological flaws, such as inadequate sample sizes, lack of proper control groups, or insufficient statistical power, which undermined their scientific rigor. Additionally, articles with unclear, insufficiently reported, or ambiguous results were excluded to ensure the reliability of the findings. Furthermore, studies focusing on tumor types other than brain tumors or specifically on the replicative mechanisms of ZIKV were excluded to keep the review focused on the effects of ZIKV on brain tumors. This approach ensured that only robust and directly applicable evidence was included.

### Process of mapping, analysis, validation, and data extraction

Following PRISMA guidelines and the Population, Intervention, Comparison and Outcome (PICO) framework [12], two authors independently examined the titles and abstracts identified in the search. Articles considered relevant were selected and downloaded for full text review. Two researchers (M.G.S.B. and B.R. M.) independently reviewed the full texts and selected articles to be included in the review based on inclusion and exclusion criteria.

Relevant characteristics of the study, including study type/design, sample size, brain tumor characteristics, evaluation parameters, intervention procedures and outcomes, were collected, analyzed, and subsequently extracted. Disagreements in data collection were discussed with the third researcher (C.VMS) until consensus was reached. Finally, a third independent researcher verified the extracted data to resolve discrepancies and verify consistency, therefore, the risk of bias for each included investigation was assessed following the guidelines of the Cochrane Collaboration Handbook [20]. When the relevant data available were limited, an attempt was made to contact the authors of the respective article to obtain the necessary data, information, and additional information.

The quality of each article was evaluated, and the level of evidence was qualified according to the classification of the Oxford Center for Evidence-Based Medicine [21].

### Risk of bias assessment

The risk of bias assessment for each study was conducted using the Cochrane Risk of Bias Tool, which evaluates various types of biases. The criteria included selection bias (random sequence generation, allocation concealment), performance bias (blinding of participants and research staff), detection bias (blinding of outcome assessment), attrition bias (incomplete outcome data), reporting bias (selective reporting), and other biases [22]. According to the Cochrane guidelines, the risk of bias was categorized into three levels: high, low, and uncertain. A high risk of bias was assigned when studies did not meet any of the assessment criteria mentioned above. Conversely, a low risk of bias was assigned when all criteria were adequately met. If the information provided was insufficient to determine the level of risk or if it was not described correctly in the article, the risk of bias was categorized as uncertain [23, 24].

## CONCLUSIONS

ZIKV therapy is promising and may reveal itself as safe and highly effective alternative to treat brain cancer. Elevated T cell activity can be used in conjunction with surgery and radiotherapy to improve survival. Despite the lack of evidence supporting the use of ZIKV for the treatment of CNS tumors in humans, the results of this review demonstrate potential benefits of this therapy in the near future. However, more rigorous clinical research is needed to validate the safety and efficacy of ZIKV in human patients. Future studies should focus on optimizing dosages, understanding the immunological mechanisms involved, and evaluating potential long-term adverse effects. Additionally, exploring the combination of ZIKV therapy with conventional treatments, such as surgery and radiotherapy, could enhance outcomes and improve patient quality of life.

## Abbreviations

CNS: Central nervous system
ZIKV: Zika Virus
GSCs: Glioma stem cells
GBM: Gliobastomas
ZVp: ZIKV prototype
OMV: Outer membrane vesicles
ZIKV-LAV: Zika virus vaccine
